# Genetic Factors Related to the Development or Progression of Mesoamerican Endemic Nephropathy

**DOI:** 10.3390/ijms26104486

**Published:** 2025-05-08

**Authors:** Alejandro Marín-Medina, Ingrid Patricia Dávalos-Rodríguez, Emiliano Peña-Durán, Luis Eduardo de la Torre-Castellanos, Luis Felipe González-Vargas, José Juan Gómez-Ramos

**Affiliations:** 1 Departamento de Biología Molecular y Genómicas, Centro Universitario de Ciencias de la Salud (CUCS), Universidad de Guadalajara, Guadalajara 44340, Jalisco, Mexico; ingriddavalos@hotmail.com; 2 Centro de Investigación Biomédica de Occidente (CIBO), Instituto Mexicano del Seguro Social (IMSS), Guadalajara 44340, Jalisco, Mexico; 3 Licenciatura en Médico Cirujano y Partero, Centro Universitario de Ciencias de la Salud (CUCS), Universidad de Guadalajara, Guadalajara 44340, Jalisco, Mexico; emilianodupe@live.com (E.P.-D.); luis.delatorre4008@alumnos.udg.mx (L.E.d.l.T.-C.); luis.gonzalez4137@alumnos.udg.mx (L.F.G.-V.); 4 Especialidad de Medicina de Urgencias Adscrita al Centro Universitario de Ciencias de la Salud (CUCS), Universidad de Guadalajara, Guadalajara 44340, Jalisco, Mexico; 5 Departamento de Urgencias, Hospital General de Zona 89, Instituto Mexicano del Seguro Social (IMSS), Guadalajara 44100, Jalisco, Mexico

**Keywords:** chronic kidney disease, chronic kidney diseases of uncertain etiology, genes, Mesoamerican nephropathy, polymorphism, genetic

## Abstract

Over the past two decades, Mesoamerican endemic nephropathy (MeN) has become a major public health problem in certain regions of Mexico and Central American countries. The etiology of this disease is multifactorial, and important environmental factors have been described, such as chronic heat stress, recurrent episodes of dehydration, infections, and exposure to toxins of chemical and biological origin. Genetic and epigenetic factors have been proposed to play significant roles in MeN. Recent studies have analyzed the role of these factors in MeN. In some cases, these factors appear to be associated with accelerated deterioration of established kidney disease due to preexisting endothelial dysfunction and tubulopathy. In other cases, they appear to be associated with early kidney damage, even before occupational exposure, suggesting that they may play a relevant role in the genesis of the disease. Other factors appear to act as risk reducers for developing MeN in areas with a high prevalence of the disease. Therefore, this disease has a rather complex multifactorial etiology, with possible polygenic contributions, possible epigenetic phenomena, and multiple environmental factors.

## 1. Introduction

Over the past 20 years, an emerging phenomenon has been observed in developing countries; young people develop a type of chronic kidney disease characterized by rapid progression and few symptoms [1].

This disease appears to have a multifactorial etiology, where the complex interaction between environmental and genetic factors is not well understood and remains understudied. Chronic Kidney Disease of uncertain or non-traditional etiology (CKDnT) is defined as a rapidly progressing type of chronic kidney disease not associated with traditional risk factors (chronic degenerative, infectious, rheumatic diseases, use of medications that cause kidney damage, etc.) and is characterized by the absence of or minimal proteinuria at the time of diagnosis [2].

A notable increase in CKDnT cases has been reported across various world regions [3]. For example, a prevalence of 1.88 per 1000 people has been reported in north-central Sri Lanka, the highest geographical prevalence recorded in that country [4]. In India, particularly in the southeastern Uddanam region, more than 34,000 people have been diagnosed with this condition, initially termed “Uddanam nephropathy”. A high incidence of the disease has also been reported in other provinces of India [5]. Over the past five decades, up to 25,000 people have been reported to be affected in countries such as Serbia, Croatia, Romania, Bulgaria, and Bosnia and Herzegovina, with an estimated 100,000 people at risk of developing the disease [6]. Although CKDnt was initially believed to be a single entity, we now recognize differences among its various forms. In the Balkan countries, this form of nephropathy is referred to as Balkan endemic Nephropathy (BeN). A genetic predisposition and a strong association with specific mutagenic agents have been identified in its etiology, while heat stress does not appear to play a significant pathogenic role [7,8].

In Latin American countries such as Mexico, Guatemala, El Salvador, Honduras, Nicaragua, Costa Rica, and Panama, cases of a form of CKDnT with characteristics similar to the nephropathy observed in India and Sri Lanka have also been reported. In the case of Asian countries, research has primarily focused on the role of toxins such as agrochemicals, heavy metals, and biological toxins in the etiology of the disease [9]. In contrast, scientific research in Latin American countries has centered on the contribution of recurrent dehydration, heat stress, and occupational exposure (particularly among male agricultural workers) to developing this CKDnT [10].

In Mexico and several Central American countries, including El Salvador, Guatemala, Honduras, Costa Rica, Panama, and Nicaragua, this condition has been termed Mesoamerican endemic Nephropathy (MeN), in reference to the broader geographic and cultural region of Mesoamerica, which is characterized by warm climates and tropical vegetation. In this context, heat stress appears to play a significant etiological role. Evidence suggests that the progressive rise in ambient temperatures in recent decades in high-prevalence areas may be directly associated with the increased incidence of this disease in these regions [11].

Although it remains unclear whether these nephropathies represent the same clinical entity, a high degree of similarity has been suggested, as both are classified as tubulointerstitial nephropathy. However, biopsies conducted on Asian patients have demonstrated a greater tendency toward interstitial inflammation and a higher prevalence of vascular and morphological alterations in the renal parenchyma. In contrast, biopsies from patients with MeN have also reported primary glomerular damage [12,13], although uncertainty persists regarding whether they represent the same clinical condition. The differences among the three forms of CKDnT are summarized in Table 1.

MeN is a tubulointerstitial nephropathy characterized by primary glomerular damage and fibrosis. It predominantly affects male agricultural workers between the second and fourth decades of life and is typically diagnosed with minimal or no proteinuria. Hydroelectrolyte disturbances, such as hyperkalemia, may be early manifestations of the disease in some individuals; however, the disease is generally asymptomatic in its early stages [18,19].

In populations in the coastal area of the Gulf of Mexico, in the south of the country, a prevalence of up to 25% of this disease has been reported [20], while in a study carried out in the area of León, Nicaragua, a prevalence of up to 35% was reported [21], and in some areas of Honduras, up to 53.9% [22].

In other countries, such as Guatemala, precise epidemiological data on the prevalence of this disease are lacking; however, it has been reported that up to 70% of hemodialysis patients are young men who are dedicated to agricultural activities [23]. In El Salvador, 2500 deaths from this disease have been reported annually, with a mortality rate of 60 per 100,000 men and 25 per 100,000 women [24]. In Costa Rica, in the Cañas and Guanacaste regions, a 13-fold increase in the mortality rate among men has been reported in recent years [25].

This indicates that there are a large number of individuals affected by this disease in Mexico and throughout Central America.

The objective of this review is to summarize the current knowledge regarding genetic factors associated with MeN, including susceptibility genes involved in the development or progression of the disease, genetic variants that may confer protection against MeN, and epigenetic factors potentially related to its onset and/or progression.

## 2. Genetic Factors Associated with the Development of Mesoamerican Endemic Nephropathy

Although genetic susceptibility appears to play an important role in the pathophysiology of MeN, its study has been limited. Although genetic susceptibility appears to play an essential role in the pathophysiology of MeN, there is little research on the genetic component of this disease. Recently, some studies have reported associations between specific genes and this disease’s development and/or progression. These studies present limitations, including the inclusion of patients from specific geographic regions within the affected countries, small sample sizes, and variability in study design. Furthermore, these studies did not assess variations in susceptibility genotypes influenced by modifier genes or environmental factors. As a result, the findings may be specific to certain populations and not generalizable to other populations or ethnic groups. Other notable limitations include the lack of studies analyzing gene–environment interactions and the absence of comprehensive genomic studies. The genetic factors described so far that have a possible role in the development of MeN are mentioned below.

### 2.1. OPCML (Opioid-Binding Protein/Cell Adhesion Molecule-like) Gene

The *OPCML* gene, located at 11q25, encodes a glycophosphatidyl-inositol (GPI)-anchored cell adhesion protein that is structurally similar to immunoglobulins. This protein plays an important role in opioid receptor signaling (particularly mu-type receptors) and requires acidic lipids for activation [26,27].

This gene has been associated with other pathologies such as ovarian cancer (due to promoter methylation) [28], progression of cholangiocarcinoma (due to loss of inactivation of the AXL/STAT3 pathway) [29], colon cancer (due to promoter hypermethylation) [30], and other types of cancer in general [31,32]; it has even been associated with the development of neuropsychiatric disorders (especially schizophrenia) through different mechanisms that mainly involve the ephrin-EphB2-cofilin pathway [33,34].

Recently, variants in this gene (rs59337101, rs73588969, and rs73588951) have been identified as potential protective factors against the development of MeN among sugarcane harvest workers in Central America.

These variants appear to confer protection against dehydration, as individuals carrying them exhibited higher plasma osmolarity under conditions of significant heat stress. This suggests that the gene may exert a regulatory effect on extracellular fluid tonicity or volume, likely through ADH (antidiuretic hormone) signaling [35,36].

Although the underlying molecular mechanisms have not been fully elucidated, some opioids are known to modulate ADH secretion, which may enhance the physiological response to hypovolemia.

Additionally, studies in mice suggest that this gene may play a role in temperature regulation, although the specific mechanisms remain unclear [33]. These findings indicate that variants in this gene may be associated with a reduced risk of developing MeN.

### 2.2. NOS3 (Nitric Oxide Synthase Type 3) Gene

The *NOS3* gene, located on 7q36, encodes the enzyme endothelial nitric oxide synthase (eNOS), which catalyzes the conversion of L-arginine to nitric oxide (NO). NO is a very important vasodilator in the kidneys that signals through the cGMP (cyclic guanosine monophosphate) pathway. It also plays an important role in other processes such as renin secretion and natriuresis [37,38].

Several polymorphisms in *NOS3*, including Glu298Asp (rs1799983), 4 b/a (VNTR in intron 4), and –786 T>C (rs2070744), have been associated with pathologies related to endothelial dysfunction, such as essential hypertension, hypertensive disorder of pregnancy (preeclampsia), pulmonary hypertension, and multiple sclerosis, among others [39,40,41,42,43]. In addition, they have been associated with other pathologies such as prostate cancer, psychiatric disorders, and osteomyelitis [44,45,46].

With regard to renal pathophysiology, the 4 b/a polymorphism has been associated with a significantly increased risk of chronic kidney disease in individuals with autosomal dominant polycystic kidney disease [47]. Furthermore, the rs2070744 variant has been identified as a potential susceptibility factor for the development and/or progression of MeN in Mexican patients under a dominant genetic model [48]. This genetic variant is associated with lower expression of the NOS3 gene (a transcription reduction of up to 50%), and studies conducted in mice with this variant showed a significant decrease in the amount of nitrites, which reaffirms the lower production of NO [49,50].

Thermal stress is the most significant environmental factor contributing to the development of NeM. It is well established that thermal stress can induce recurrent episodes of dehydration, leading to increased uric acid concentrations in the renal tubules [51,52,53]. This, in combination with a substantial reduction in NO, may result in a diminished anti-inflammatory response and vasoconstriction of the afferent and efferent renal arterioles. These changes can alter renal hemodynamics and impair the regulation of natriuresis, potentially accelerating the progression of kidney disease in affected individuals [54].

### 2.3. NGAL (Neutrophil Gelatinase-Associated Lipocalin) Gene

The *NGAL* gene, located on 9q34, encodes a protein belonging to the lipocalin family, which performs multiple biological functions, including the regulation of bacterial growth through siderophore sequestration and the transport of lipid molecules [55]. In the renal context, *NGAL* can form complexes with siderophores to chelate iron released during tubular epithelial injury, thereby mitigating oxidative stress [56].

This protein is a biomarker traditionally related to acute kidney injury, but it has also been reported to be associated with essential hypertension, obesity, cardiovascular diseases, and even cancer [57].

Although genetic variants of *NGAL* associated with MeN have not been studied to date, a study was conducted in adolescents from different regions of Nicaragua with no history of agricultural occupations and in regions with a high prevalence of MeN. Concentrations of this protein were measured, and a significant increase in its concentration was observed in these individuals (mean of 13.3 ng/mg), which could indicate a probable role of this gene in early kidney damage before occupational exposure [58]. This finding supports the hypothesis that *NGAL* may contribute to the early pathogenesis of MeN through mechanisms related to oxidative stress regulation at the level of renal tubules. However, studies that delve into the possible role of genetic variants of *NGAL* in the development of MeN are needed, since it could have a relevant participation in the regulation of oxidative stress in the renal tubules.

### 2.4. ApoE (Apolipoprotein E) Gene

This gene, located at 19q13.32, encodes apolipoprotein E and is essential for lipoprotein catabolism in the liver. In addition to its role in lipid metabolism, it has a very important effect on vascular smooth muscle in arterioles and the regulation of mesangial cell proliferation (which, once altered, can cause injury to podocytes and renal tubular cells). Therefore, genetic variants of ApoE could contribute to kidney injury [59,60].

The *APOE4* variant has been associated with death by suicide and neurodegenerative diseases in the Mexican population [61], while the *APOE2* variant has been associated with the development of diabetic nephropathy [62].

In a recent study conducted in a Panamanian population with MeN, it was found that the rs429358 variant (p. C136R) of this gene was associated with hyperuricemia [63], which may contribute to the development of endothelial dysfunction and tubulopathies. In this sense, repeated dehydration episodes in patients carrying this genetic variant would drastically increase uric acid levels, and this could contribute to the development or progression of MeN [64].

It has already been described that uric acid in serum has a somewhat relevant antioxidant effect [65], but when it enters the cell, uric acid can contribute to the peroxidation of lipids, proteins, and nucleic acids and can also stimulate the production of proinflammatory cytokines such as tumor necrosis factor alpha (TNF-α) [66,67].

In this context, it can be said that hyperuricemia seems to contribute through several mechanisms to tubular injury and endothelial dysfunction in individuals with MeN.

Genetic factors possibly associated with MeN are summarized in Table 2.

## 3. Epigenetic Factors Related to MeN

### 3.1. Role of Differentially Methylated Regions (DMRs) in the Development of MeN

DMRs are genomic regions with distinct methylation patterns in different samples that regulate the expression of flanking genes. Therefore, alterations in the methylation patterns of some of these regions on some chromosomes have been associated with various diseases [68].

#### 3.1.1. DMR on Chromosome 7

##### *AMPH* (Amphiphysin 1) Gene

The *AMPH* gene located on 7p14.1 encodes the protein Amphiphysin 1; this protein performs functions related to vesicular trafficking in endocytosis and receptor recycling at the synapse [69].

Reduced AMPH mRNA levels have been reported to be significantly associated with patients with advanced breast cancer, as well as with metastasis and an inadequate response to treatment [59]. Furthermore, antibodies against this protein are known to form in some autoimmune diseases such as Stiff-Person syndrome and limbic encephalitis [70,71].

In a recent study evaluating the relevance of DMRs in the development of MeN, the DMR on chromosome 7, which regulates *AMPH* gene expression, was found to be associated with the development of MeN [72]. Furthermore, it was observed that changes in methylation patterns, associated with exposure to pesticides and heavy metals, were not related to new or already identified cases of this disease [72], indicating that exposure to this type of toxin might not be the triggering factor of the disease and reinforcing the hypothesis of the existence of other environmental factors, such as dehydration and personal thermal stress, which may act as triggering elements in the etiology of this disease.

Although the role of this gene in MeN remains uncertain, it is known to play a fundamental role in cellular signaling through various pathways, including Ras/GDP/GTP and RAB5/GAP/TCB-2, among others. These pathways are essential for processes such as signal transduction, endosome formation, and receptor recycling [73,74]. Therefore, alterations in the imprinting of the DMR on chromosome 14, which regulates the expression of this gene, may lead to significant disruptions in key cellular signaling processes at the renal level in individuals with MeN.

#### 3.1.2. DMR on Chromosome 10

##### *SLC29A3* (Solute Carrier Family 29 “Nucleoside Transporters Member 3”) Gene

This gene, located at 10q22.1, encodes a protein called ENT3, which is involved in the transport of nucleosides, nucleotides, and nucleoside drugs, as well as in signal transduction processes [75].

Mutations in this gene cause H syndrome, an autosomal recessive disorder characterized by hypertrichosis, hyperpigmentation, hepatomegaly, cardiac abnormalities, and hypogonadism, among other manifestations [76].

The DMR regulating the expression of this gene has not been associated with kidney disease of any etiology, so it may be unique to MeN [72].

Although the biological importance of this DMR in MeN is still unclear, a recent study that evaluated renal metabolomics in the context of MeN found an alteration in NAD+ metabolism, partly due to a decrease in the activity of the enzyme quinolinate phosphoribosyltransferase, a key enzyme in the synthesis of this compound from tryptophan (since elevated urinary levels of tryptophan/kynurenate were found in MeN), although this appears to be the main pathway for the formation of NAD+ at the renal level; there are two other pathways for NAD+ synthesis, involving nicotinic acid and the salvage pathway using nicotinamide [77,78]. Therefore, this alteration in mitochondrial energy metabolism could contribute to several mechanisms of kidney damage, and especially to fibrogenesis [79]. Although the mechanism of transport of these compounds at the renal level is not entirely clear, the *SLC29A3* gene could participate in the transport of compounds of this metabolic pathway at the renal level. When there are alterations in this gene or in the DMR that controls its expression, it could contribute to a more significant depletion of NAD+ in the context of MeN.

#### 3.1.3. DMR on Chromosome 14

This region is particularly important, as it is associated only with individuals with established kidney disease and, therefore, with severe kidney function impairment [72].

##### *DIO3* (Iodothyronine Deiodinase 3) Gene

This gene, located at 14q32.31, encodes a selenoprotein that catalyzes the deiodination of T3 and T4 to inactive metabolites [80]. Alterations in the imprinting of this DMR have been associated with diseases such as cancer, developmental disorders, and respiratory diseases [80,81].

T3 transport in the kidney is mediated by several proteins, including MTC8, MTC10, and LAT2. *DIO3* appears to have a direct effect on renal T3 concentrations and is required for the function of MTC8 [80].

T3 has several effects on the kidney. For example, it is essential for the expression of Na^+^/K^+^ ATPase. This enzyme maintains the electrogenic potential of many cells and is responsible for many secondary transport mechanisms, such as Na^+^/H. It regulates renin expression and increases the activity of angiotensin-converting enzyme (ACE). It also regulates angiotensin 1 and 2 receptors. It has several effects on endothelial cells, where it can induce vasodilation independently of NO signaling and also appears to have a proangiogenic effect [82,83,84,85]. Therefore, alterations in the DMR that controls the expression of this locus could lead to an increased tendency toward vasoconstriction of glomerular arterioles, modifications in the electrogenic potential of cells (which could cause the failure of several cotransport mechanisms in the kidney), and alterations in the function of endothelial cells, which together could cause kidney damage. A decrease in glomerular filtration rate and even kidney injury have already been described in patients with hypothyroidism, so a prolonged hypothyroid state could contribute to irreversible kidney injury [86,87].

##### *RTL1* (Retrotransposon Gag Like 1) Gene

This gene, located at 14q32.2, has a relevant role in the function of the endothelial cells of the capillaries of the fetus and the placenta [88] and seems to have a significant role in angiogenesis [89]. Studies in mice have reported that this gene plays an important role in regulating ion channels in the nervous system [90].

The expression of this gene is controlled by the DMR on chromosome 14. It is a monoallelic gene expressed from the paternal allele. Alterations of this DMR in the context of MeN have been associated with established kidney disease [72].

Although the precise role of this gene in MeN is unknown, experiments in mice have demonstrated that it is essential for maintaining the structure and function of capillaries in various tissues, likely through interactions with other genes such as *PEG10* [91]. Consequently, this gene may play a significant role in the integrity of renal capillaries.

##### *DLK1* (Delta Like Non-Canonical Notch Ligand 1) Gene

This gene, located at 14q32.2, encodes a protein that plays an important role in cell growth and proliferation (through the NOTCH pathway), particularly in adipogenesis. It also appears to play an important role in cell regeneration; however, the related molecular mechanisms are poorly understood [92]. In the context of kidney disease, this gene has been observed to act as a NOTCH antagonist and a modulator of the immune response [93]. Although the role of this gene in NeM is uncertain, it is known that disruptions at various points along this pathway, particularly in the context of diabetic nephropathy, can lead to tubulointerstitial fibrosis, endothelial dysfunction, podocyte injury, and activation of inflammatory pathways, among other pathological changes [94]. Furthermore, this *DIO-DLK1* imprinting center has been associated with reduced estimated glomerular filtration rate in Latino, Hispanic, and African American individuals [95].

Therefore, alterations in this gene may contribute to an exacerbated inflammatory response, tubular fibrosis, and endothelial dysfunction in the context of NeM. However, further studies examining the genes regulated by the DMR on chromosome 14 are needed to clarify their precise role in the pathogenesis of NeM.

The role of DMRs in MeN is summarized in Table 3.

## 4. Possible Molecular Mechanisms Involving Genetic Factors Associated with MeN

In the case of the *NGAL* gene, its expression product is important for the formation of siderophore complexes that prevent ferroptosis of tubular cells, which could prevent tubulopathy [56,58].

When eNOS enzyme expression decreases, NO production is significantly reduced, which can lead to endothelial dysfunction and a decreased anti-inflammatory response (factors that may contribute to tubulointerstitial injury and rapid decline in kidney function) [37,38,49]. In this regard, *APOE* gene variants have been associated with hyperuricemia, which, together with repeated episodes of dehydration due to personal heat stress, could cause tubulointerstitial injury. Furthermore, uric acid could penetrate tubular cells and cause lipid and protein peroxidation, as well as a considerable increase in the activation of proinflammatory pathways [59,64].

When there are alterations in the imprinting of the DMR that controls the expression of the *SLC29A3* gene, the functioning of the ENT3 protein (which acts as a transporter of nucleotides and nucleosides) could be altered, which could contribute to a decrease in the concentration of NAD+ in the tubular cells, which could cause a depletion of this compound in the kidney and lead to serious metabolic alterations due to a decrease in the transport of electrons to the mitochondrial respiratory chain and, consequently, a collapse in energy metabolism [77,78,79]. In that sense, alterations in the imprinting region of chromosome 14 (which controls the expression of the *DIO3* gene) could affect the transport of the T3 hormone to the kidneys. This would affect active and passive transport mechanisms, ultimately affecting the homeostasis of the electrogenic potential in these cells and potentially contributing to the development of tubular fibrosis [82,83]. Another gene controlled by this DMR is *DKL1*, which could alter the regulation of the NOTCH1 pathway, which controls key processes such as cell proliferation and differentiation, angiogenesis, and others [92,93]. Therefore, there are various molecular mechanisms that could be related to the development or progression of MeN, which, together or individually and in interaction with various environmental factors, could contribute to tubulopathy and progressive renal deterioration.

**Figure 1:**
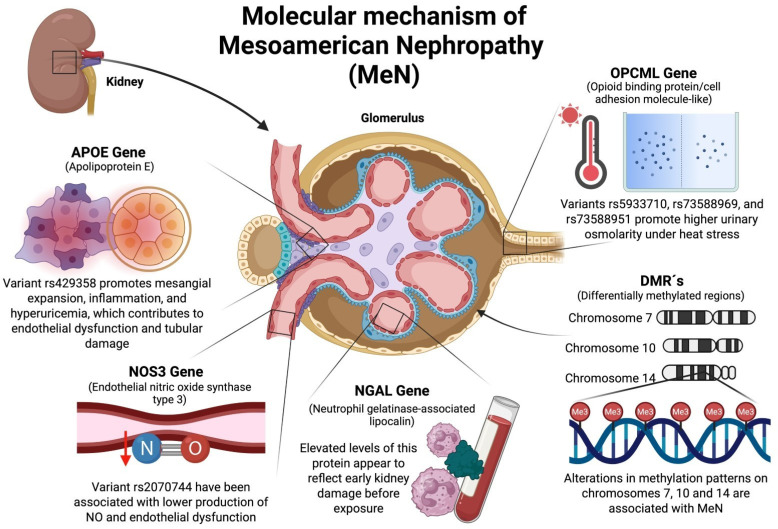
Proposed molecular mechanisms associated with genetic susceptibility to Mesoamerican Nephropathy (MeN). This schematic illustrates the role of several candidate genes and differentially methylated regions (DMRs) implicated in the development and progression of MeN. Variants in the *OPCML* gene (rs5933710, rs73588969, rs73588951) have been associated with increased plasma osmolality under heat stress, suggesting a protective role via extracellular fluid regulation. The *NGAL* gene, a biomarker for early tubular injury, shows elevated protein expression even in individuals without occupational exposure, indicating possible early renal stress. *NOS3* (rs2070744) and *APOE* (rs429358) variants have been linked to reduced nitric oxide production, endothelial dysfunction, hyperuricemia, and mesangial inflammation, contributing to tubulointerstitial damage. Epigenetic alterations in DMRs located on chromosomes 7 (*AMPH*), 10 (*SLC29A3*), and 14 (*DIO3, DLK1, RTL1*) have also been associated with MeN, potentially modulating pathways related to mitochondrial energy metabolism, thyroid hormone signaling, and endothelial integrity.

Possible molecular mechanisms related to the development or progression of MeN are shown in Figure 1.

## 5. Conclusions

There are few studies examining the genetic factors associated with the development or progression of MeN, as most research has focused on environmental contributors. Variants in *NOS3* and *APOE*, and the DMR on chromosome 14, appear to be associated with established kidney disease and may play a central role in the accelerated decline of kidney function in MeN. In this context, the *NGAL* gene and the DMRs on chromosomes 7 and 10 have been linked to the disease’s onset, further supporting the hypothesis that individual susceptibility to heat stress and dehydration are primary environmental factors in the etiology of MeN. Additionally, variants in *OPCML* have been associated with a lower risk of developing the disease. These findings could inform a classification system for genetic factors based on their association with increased or decreased risk, as well as with the rate of disease progression or other, yet unidentified, subtypes. However, genome-wide association studies (GWAS), investigations of gene-environment interactions, epigenetic analyses, large-scale prospective cohort studies, and metabolomics research are still needed. Future studies should also assess gene expression under conditions of heat stress and explore how susceptibility genotypes may vary in effect due to the presence of primary or secondary modifier genes and environmental influences.

## Figures and Tables

**Table 1:** Differences between the main types of CKDnT. BeN (Balkan endemic Nephropathy), MeN (Mesoamerican endemic Nephropathy).

Type of CKDnT	Geographical Area of High Prevalence	Main Etiological Agents	Main Histopathological Characteristics
BeN	Serbia, Croatia, Bosnia and Herzegovina, Romania, and Bulgaria	Exposure to aristolochic acid, genetic predisposition identified [14].	Tubulointerstitial nephropathy with fibrosis. Secondary glomerular damage, no chronic vascular alterations are observed [15].
Uddanam nephropathy	India and Sri Lanka	Exposure to pesticides, heavy metals, and other chemical and biological agents. Possible genetic predisposition [5].	Tubulointerstitial nephropathy with fibrosis. No primary glomerular damage is observed; secondary vascular alterations are present [16,17].
MeN	Mexico, Guatemala, El Salvador, Honduras, Nicaragua, Costa Rica, and Panama	Occupational exposure to high temperatures, contamination with silica particles. Possible genetic predisposition [10].	Tubulointerstitial nephropathy with fibrosis, which may be accompanied by primary glomerular damage and secondary vascular alteration [17].

**Table 2:** Genetic factors related to the development or progression of MeN. *OPCML* (Opioid binding protein/cell adhesion molecule-like), *NOS3* (Nitric Oxide Synthase type 3), *NGAL* (Neutrophil gelatinase-associated lipocalin), *APOE* (Apolipoprotein E), NO (Nitric Oxide).

Gene	Chromosomal Location	Possible Relationship with MeN
*OPCML*	11q25	Variants in this gene may be associated with a lower risk of MeN. This gene appears to play a role in regulating temperature.
*NOS3*	7q36	The rs2070744 variant of this gene is associated with decreased NO production, so this variant appears to be related to endothelial dysfunction and possibly glomerular damage, which could contribute to the rapid progression of the disease.
*NGAL*	9q34	It appears to be associated with early kidney damage prior to occupational exposure in areas of high MeN prevalence.
*APOE*	19q13	The rs429358 variant appears to be associated with hyperuricemia (a previously described risk factor for the development of this disease); therefore, this variant appears to be linked to accelerated disease progression.

**Table 3:** Possible role of DMRs in the development or progression of NeM. DMR (Differentially Methylated Region), *AMPH* (Amphiphysin 1), *SLC29A3* (Solute Carrier Family 29 “Nucleoside Transporters Member 3”), *DIO3* (Iodothyronine Deiodinase 3), *RTL1* (Retrotransposon Gag Like 1), *DLK1* (Delta Like Non-Canonical Notch Ligand 1).

Chromosomal Location of DMRs	Regulated Genes	Role in the Development or Progression of MeN
7p14	*AMPH*	This DMR has not been previosuly associated with kidney disease, so it may be unique to MeN and could play an important role in the genesis of disease. The genes regulated by this region perform functions related to vesicular trafficking and receptor recycling.
10q22	*SLC29A3*	This DMR has not been previosuly associated with kidney disease, so it may be unique to MeN and could play an important role in the genesis of disease. The genes it regulates play a central role in the transport of high-energy molecules (NAD+ and analogues) in tubular cells.
14q32	*DIO3* *RTL1* *DLK1*	The DMR that controls the expression of these genes appears to be associated with established and rapidly progressing kidney disease, since the genes controlled by this region have functions related to angiogenesis, endothelial function, cell growth and proliferation.
